# High-frequency percussive ventilation in acute respiratory distress syndrome: knocking at the door but can it be let in?

**DOI:** 10.1186/s13054-018-1982-4

**Published:** 2018-03-02

**Authors:** Herbert Spapen, Jouke De Regt, Viola van Gorp, Patrick M. Honoré

**Affiliations:** 1 Intensive Care Department, University Hospital, Vrije Universiteit Brussel (VUB), Laarbeeklaan 101, B-1090 Brussels, Belgium; 2 Intensive Care Department, Centre Hospitalier Universitaire Brugmann, Brussels, Belgium

High-frequency ventilation has been proposed as an alternative ventilation mode for acute respiratory distress syndrome (ARDS). Most experience was gained with high-frequency oscillation but this technique became abandoned due to unwarranted side effects (higher need for sedation and neuromuscular blockade, prolonged hemodynamic instability) and a higher mortality [1].

Godet et al. [2] recently documented the effects of short-term application of high-frequency percussive ventilation (HFPV) in animals and patients with early nonfocal moderate to severe ARDS. HFPV highly improved oxygenation and hemodynamics. In addition, HFPV allowed significant alveolar recruitment without concomitant hyperinflation of aerated lung regions [2]. Based on their observations, the authors suggested further investigation of HFPV on patient outcome during ARDS.

We have published the largest observational study to date on clinical use of HFPV in ARDS [3]. We retrospectively analyzed data from 42 patients (20 pneumonia-induced cases and 22 pneumonia-unrelated cases) with moderate to severe ARDS. HFPV was governed according to a predefined protocol and continued until patients could be switched to conventional ventilation. Essentially, our results corroborate the findings of Godet et al. [2]. Within 24 h, oxygenation improved to a similar proportion (i.e., doubling of the PaO_2_/FiO_2_ ratio). PaCO_2_ was kept normal, barotrauma never occurred, and no significant hemodynamic changes were observed during the course of HFPV treatment. Moreover, the respiratory and hemodynamic benefits were sustained for at least 6 days after initiation of HFPV. Interestingly, less improved oxygenation, longer ventilation and ICU dependency, and higher 30-day mortality (50% vs 18%; *P* = 0.01) were observed in pneumonia-related ARDS. Most deaths in the pneumonia group were related to intractable multiorgan failure. Whether HFPV adversely propagates reactive pathways in pneumonia-related ARDS that ignite remote inflammation and sustain organ damage remains unknown.

According to Godet et al. [2], HFPV perfectly fits within the “Open the Lung and Keep it Open” concept of protective lung ventilation in ARDS. Applying HFPV, however, differs considerably from a low tidal volume/high positive end-expiratory pressure (PEEP) ventilation strategy where personalizing the PEEP level is primordial to minimize dynamic strain caused by alveolar recruitment/derecruitment [4]. Conventional HFPV settings may deliver injurious tidal volumes [5]. HFPV also significantly interferes with sedation protocols (e.g., low-level sedation, sedation breaks, etc.) and prone positioning, and requires supervision on a 24/7 basis by a dedicated team of trained physicians and respiratory therapists. Our clinical experience with HFPV does not support current introduction of this technique for ventilating ARDS patients.

## Abbreviations

ARDS: Acute respiratory distress syndrome
HFPV: High-frequency percussive ventilation
PEEP: Positive end-expiratory pressure
